# Modified Spraying Technique and Response Surface Methodology for the Preparation and Optimization of Propolis Liposomes of Enhanced Anti-Proliferative Activity against Human Melanoma Cell Line A375

**DOI:** 10.3390/pharmaceutics11110558

**Published:** 2019-10-28

**Authors:** Hesham Refaat, Youssef W. Naguib, Mahmoud M. A. Elsayed, Hatem A. A. Sarhan, Eman Alaaeldin

**Affiliations:** 1 Department of Pharmaceutics, faculty of Pharmacy, Minia University, Minia 61519, Egypt; Hesham.refaat@deraya.edu.eg (H.R.); youssefwahibnaguib-ibrahim@uiowa.edu (Y.W.N.); ha_sarhan@yahoo.com (H.A.A.S.); 2 Department of Pharmaceutics, faculty of Pharmacy, Deraya University, Minia 61111, Egypt; 3 Division of Pharmaceutics and Translational Therapeutics, College of Pharmacy, University of Iowa, Iowa City, IA 52242, USA; 4 Department of Pharmaceutics and Clinical Pharmacy, Faculty of Pharmacy, Sohag University, Sohag 22161, Egypt; mahmoudalmenshawy@pharm.sohag.edu.eg; 5 Department of clinical Pharmacy, faculty of Pharmacy, Deraya University, Minia 61111, Egypt

**Keywords:** propolis, liposomes, spraying technique, response surface methodology, enhanced cellular uptake

## Abstract

Propolis is a honeybee product that contains a mixture of natural substances with a broad spectrum of biological activities. However, the clinical application of propolis is limited due to the presence of a myriad of constituents with different physicochemical properties, low bioavailability and lack of appropriate formulations. In this study, a modified injection technique (spraying technique) has been developed for the encapsulation of the Egyptian propolis within liposomal formulation. The effects of three variables (lipid molar concentration, drug loading and cholesterol percentage) on the particle size and poly dispersity index (PDI) were studied using response surface methodology and the Box–Behnken design. Response surface diagrams were used to develop an optimized liposomal formulation of the Egyptian propolis. A comparative study between the optimized liposomal formulation prepared either by the typical ethanol injection method (TEIM) or the spraying method in terms of particle size, PDI and the in-vitro anti-proliferative effect against human melanoma cell line A375 was carried out. The spraying method resulted in the formation of smaller propolis-loaded liposomes compared to TEIM (particle sizes of 90 ± 6.2 nm, and 170 ± 14.7 nm, respectively). Furthermore, the IC50 values against A375 cells were found to be 3.04 ± 0.14, 4.5 ± 0.09, and 18.06 ± 0.75 for spray-prepared propolis liposomes (PP-Lip), TEIM PP-Lip, and propolis extract (PE), respectively. The encapsulation of PE into liposomes is expected to improve its cellular uptake by endocytosis. Moreover, smaller and more uniform liposomes obtained by spraying can be expected to achieve higher cellular uptake, as the ratio of liposomes or liposomal aggregates that fall above the capacity of cell membrane to “wrap” them will be minimized.

## 1. Introduction

Natural products have caught the attention of formulators to design a suitable dosage to maximize the therapeutic effects of these effective components. Propolis (also known as bee glue) is a resinous substance collected by honeybees from different plant parts, such as barks, cracks and leaf buds. Due to its strong adhesive properties, bees use propolis in the establishment and protection of their hives [1,2]. Propolis has a unique chemical composition as it is rich in polyphenolics, flavonoids, resins, balsams, waxes, amino acids and oils [3]. Many studies jave investigated the pharmacological activity of propolis [4,5,6,7,8,9], including its anticancer [10], antioxidant [11], antimicrobial [12], antiviral [13] and wound healing [14] properties, in addition to immunomodulatory effects [15]. Propolis has been reported to possess cytotoxic activity against different murine and human melanoma cell lines, including B16-F10 [16], B16-F1, and A375 [17]. Although propolis has an expansive therapeutic potential, its processing and formulation development are strictly hindered due to its resinous and sticky consistency, low solubility, and physical instability [18]. 

Liposomes are nano-sized lipid-based vesicles composed of a lipid bilayer (composed mainly of phospholipids) surrounding an aqueous core [19]. Liposomes are capable of entrapping different active pharmaceutical ingredients such as antimicrobial and antineoplastic drugs, chelating agents, vaccines, steroids and genetic material [20]. Liposomes can be modified using several methods to increase their stability via freeze drying [21,22], surface polymerization [23], surface coating [24] or other processes. They have a unique architecture with their nanoscale, biocompatible, biodegradable and flexible characters which offer a controlled drug delivery to specific organs [25]. Size is the most crucial factor in the in vivo behavior of liposomes and controls the penetration and targeting of drug-loaded vesicles [26]. However, many of the preparation techniques of liposomes produce a heterogenous population, and liposomes may contain residuals of organic solvents [27]. Liposomes larger than 100 nm are more prone to opsonization and clearance by the reticuloendothelial system in high rates than smaller ones [28]. Many techniques have been applied to control the size and homogeneity of liposomal preparations. Sonication is an example of a size-reducing technique [29] which may damage the phospholipids due to the energy input and liability of oxidation [30]. Another technique is the extrusion process [31], which involves using expensive, time-consuming and sensitive devices [32,33,34]. 

In this study, a simple, new and efficient modification of the ethanol injection method is introduced for the production of homogenous, nanosized liposomes with the avoidance of the drawbacks of other size-controlling techniques. The prepared liposomes were used for the entrapment of Egyptian propolis with its pharmacologically active flavonoids (galangin and chyrsin [35]). A comparative study was carried out to estimate the efficiency of the modified method (spraying technique) in the preparation of homogenous, smaller nanosized liposomes compared to the typical ethanol injection method (TEIM). We hypothesize that a smaller uniform nanosized form of propolis (prepared using a spraying technique and proposed here) will improve its anti-proliferative activity against A375 melanoma cells.

## 2. Experimental

### 2.1. Materials

Lipoid S75 (fat-free soybean phospholipids with 70% phosphatidylcholine) was a gift from the lipoid company (Ludwigshafen, Germany). Propolis extract (PE) was purchased from Cell Culture Department-VACSERA-EGYPT. Cholesterol was purchased from Fluka Chemical Co. (Kolkata, India). Absolute ethanol was purchased from El-Nasr Pharmaceuticals (Cairo, Egypt). Distilled water was supplied by the analytical chemistry laboratory, Deraya university. A375 human melanoma cell line cells were obtained from the American Type Culture Collection; cells were cultured using DMEM (Invitrogen/Life Technologies) supplemented with 10% FBS (Hyclone), 10 μg/mL of insulin (Sigma, St. Louis, MO, USA), and 1% penicillin–streptomycin. MTT assay kits were purchased from Sigma Aldrich. All of the other chemicals and reagents were of analytical grade (Sigma Aldrich, St. Louis, MO, USA).

### 2.2. Methodology

#### 2.2.1. Spraying Technique for the Preparation of PP-Lip 

Propolis liposomes (PP-Lip) were prepared using a new modified injection method. Briefly, lipoid s75, cholesterol and propolis were dissolved in the least volume of absolute ethanol; then, the solution was transferred to a spraying apparatus. In a closed system, the solution of lipids and propolis was sprayed (200 µL per five seconds) on the surface of an aqueous media of distilled water containing sucrose (9% *w*/*v*) stirred at 1500 rpm at 80 °C. Excess ethanol was evaporated with stirring, and the liposomes were formed spontaneously after further evaporation of the residual ethanol. Prepared liposomes were kept at 4 °C overnight to allow the annealing of the lipid bilayer [36]. The unencapsulated propolis aggregated at the surface of the aqueous liposomal suspension upon cooling and was manually removed by means of a clean wood scraper. The liposomal suspension was then available for the further processes of evaluation and in-vitro characterization. The particle size and poly dispersity index (PDI) were determined, and transmission electron microscopy (TEM) was used to evaluate the liposomes.

Liposomal size and size distribution were determined using laser light diffraction techniques. Briefly, the liposomal preparation was diluted using purified deionized water and analyzed at 25 °C using a Mastersizer 3000E (Malvern Instruments, Malvern WR14 1X, UK). This procedure was done in triplicate for each preparation, and the average values were used.

Transmission electron microscope images were taken after a dilation of the sample with distilled water, and the sample was allowed to air dry on a carbon grid for 1 h and then examined with JEOL (JM 1000 EX, Peabody, MA, USA).

#### 2.2.2. Entrapment Efficiency Percentage (%EE) of Prepared Liposomes

The percentage of flavonoidal content entrapped within the prepared liposomes was determined as follows; first, the free drug was separated from liposomes by the centrifugation of 1 mL of different liposomal suspensions at 15,000 rpm for 2 h at 4 °C. The separated liposomes were washed twice in two separate steps by resuspending them in distilled water to ensure the absolute removal of the unentrapped drug. Then, the liposomes were exposed to absolute alcohol followed by vortexing and sonication until a homogenous suspension was obtained. The suspension was then centrifuged at 15,000 rpm for 30 min, and the supernatant was separated. The colorimetric analysis of the flavonoid content of propolis was previously reported by Woisky and Salatino [37]. One hundred microliters of 10% aluminum chloride alcoholic solution was added to 100 µL of the supernatant, and absolute alcohol was added to obtain a final volume of 2 mL; then, the absorbance was measured at 410 nm using a UV/Vis spectrophotometer (Spectronic Genesys^®^, with Winspec Software, Spectronic, (Pittsford, NY, USA). The blank was prepared for the direct method using the same procedures as drug-free (blank) liposomes, and its absorbance was then subtracted from that of the propolis-loaded liposomes. Steps were repeated in a triplicate manner, and the mean of flavonoid entrapment was determined using the following equation:

$$E.E\;\%\;=\;\frac{\;\left(amount\;of\;flavonoids\;entrapped\right)}{\left(total\;amount\;of\;flavonoids\;added\;\right)\;}\;\times \;100$$

#### 2.2.3. Optimization of Preparation Parameters of PP-Lip Using Response Surface Methodology (RSM), and Box–Behnken Design of Preparation Conditions

Three important factors were selected to study and optimize their effect on the particle size and PDI of the prepared liposomes. Lipid molar concentration (LMC), cholesterol percentage (CH%) and flavonoid loading—which can be metaphorically expressed as drug loading (DL)—were used at three levels, and 17 experiments were generated according to the Box–Behnken design. The three levels of the three factors were as follows: LMC (40, 60, 80 mmole), CH% (20, 43, 66%), and DL (1.5, 3.25, 5 mg) (Table 1).

#### 2.2.4. RSM Approach for Optimization of Factors

According to the RSM approach, the runs were applied in Box–Behnken model-designed experiments to approve that the effects of independent factors were coherent in both the regression model and the experimental results. The response surface diagram was constructed using Design Expert software, version 11 (StatEase^®^, Minneapolis, MN, USA). Formulations were optimized using response surface diagrams.

#### 2.2.5. Spraying Technique Versus TEIM

Based on the RSM approach optimization results, the optimized PP-Lip formulation was prepared by both the spraying technique, as previously described, and the TEIM, as prepared by Yuan et al. [38]. Briefly, the lipids and propolis were dissolved in 10 mL absolute ethanol and directly injected into a buffer with a slow speed and thermostatic mixing. The particle size and PDI were measured for the two prepared formulations for the assessment of the efficiency of the new modification in producing smaller nanosized liposomes. EE% was calculated for the two preparations.

#### 2.2.6. In Vitro Cytotoxicity Assay

To study the effect of the spraying technique on the enhancement of the cytotoxic effect of the PE against human melanoma cell line A375, the in vitro cytotoxicity was determined using MTT assay. Briefly, cells were seeded in 96-well plates at an initial density of 2 × 10^3^ cells/well. After 24 h, the prepared liposomes as well as the PE were diluted with medium to prepare five concentrations of each sample (100, 25, 6.25, 1.56 and 0.39 µg propolis/mL). Cells were treated with either TEIM PP-Lip, spraying prepared liposomes or PE. Both drug-free (blank) liposomes and the extracted solvent of propolis were used as controls to eliminate the cytotoxic effect of the lipids and solvent. After incubation at 37 °C for 72 h, MTT reagent was added (50 μL of 5 mg/mL MTT solution in DMSO), and cells were further incubated at 37 °C for 4 h. The absorbance of each well was read at 450 nm using a microplate reader (Bioline Elisa plate reader, Maharashtra, India) according to the manufacturer’s instructions. Data are representative of three independent experiments.

All experiments were carried out in triplicate. GraphPad Prism 8 (GraphPad Software, San Diego, CA, USA) was used to analyze the data. One-way ANOVA followed by Tukey’s post-hoc test was used to calculate the significance of the recorded differences. A *p* value of less than 0.05 was considered statistically significant.

## 3. Results 

### 3.1. Preparation of PP-Lip

**Figure 1:**
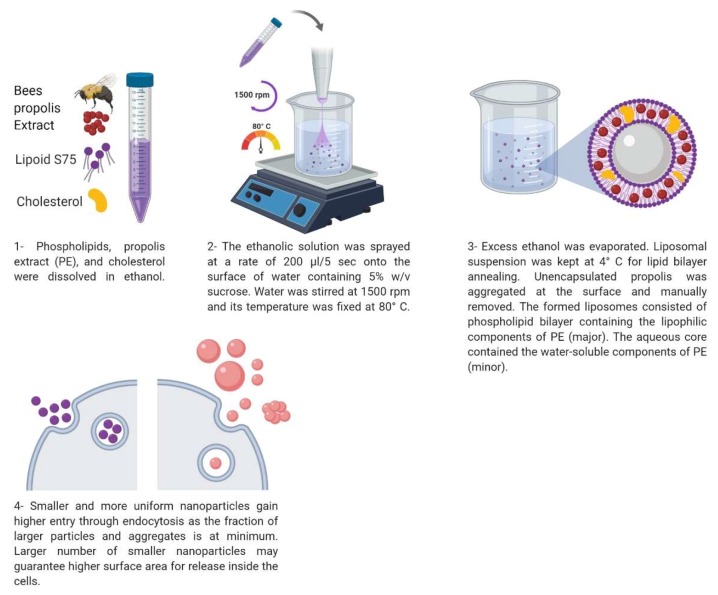
Schematic diagram showing the details of the spray based preparation method of PP-Lip. This figure was created using Biorender.com.

**Figure 2:**
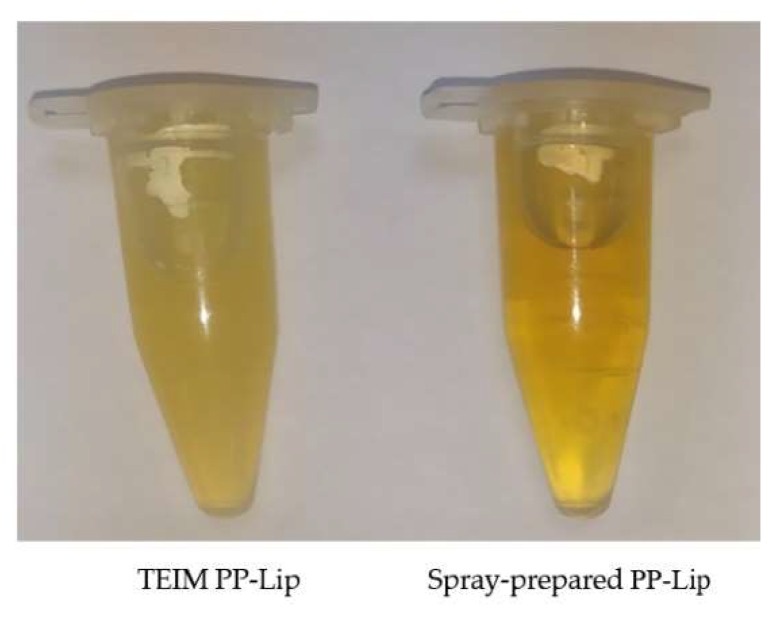
Macroscopic examination of the typical ethanol injection method (TEIM)-prepared and spray-prepared PP-Lip.

The spraying method successfully produced a nanosized uniform propolis-loaded liposomal formulation (Figure 1), with a very minor aggregation of unencapsulated propolis during preparation and a neat macroscopic appearance of the prepared liposomes (Figure 2), with high propolis entrapment (57.43 ± 0.09%).

### 3.2. Response Surface Methodology (RSM) Approach for the Optimization of Preparation Factors 

The effects of three independent factors—LMC, CH%, and DL—on the particle size and PDI of the produced PP-Lip were estimated for the 17 runs. The measurement of the particle size and PDI were determined in three replicates, and the average has been calculated (Table 2). 

Measured data were analyzed using Design Expert software. The regression equations derived by the model were as follows:

Y_F_ = X_o_ + X_1_A + X_2_B + X_1_X_1_ A_1_A_1_+X_2_X_2_ B_2_B_2_

where Y_F_ is the independent variable, X_o_ is the arithmetic mean response for the 17 runs, and X_1_ is the estimated coefficient factor for A. A and B represent the average of changing one dependent variable at one time. A_1_A_1_ and B_2_B_2_ are used to investigate non linearity. The sign and magnitude of coefficients indicates the effectiveness of dependent variables on the response.

ln (Particle size) = +5.45 + 1.21 A + 0.4435 B − 0.2401 C + 0.2246 A^2^ + 0.1657 B^2^ + 0.3924 C^2^

PDI = +0.3806 + 0.2445 A + 0.2178 B + 0.0015 C + 0.0698 A^2^ + 0.1298 B^2^ + 0.0288 C^2^

### 3.3. Optimized Formulation Parameters for the Lowest Particle Size

The particle sizes of performed batches of liposomes ranged between 80.6 and 1755 nm. The verified model for the particle size is

ln (Particle size) = +5.45 + 1.21 A + 0.4435 B − 0.2401 C + 0.2246 A^2^ + 0.1657 B^2^ + 0.3924 C^2^

The variance analysis of the regression model for the particle size of PP-Lip was checked by the *p*-value and *F*-test (Table 3). The *p*-value was highly significant (*P* ˂ 0.0001) and the lack-of-fit *p*-value was larger than 0.05 (0.1562), which indicates that the fitted regression equation was good and capable of explaining and predicting the results. Table 3 showed that the individual variables of LMC (A), CH% (B), DL (C) and (C^2^) (the quadratic variable of DL which represents an excess of DL) are significant model terms. The positive signs of the coefficients of LMC and CH% indicate the increased particle size with increasing LMC and CH%. The negative sign of the DL coefficient indicates that increasing DL decreases the particle size of formulated liposomes. The coefficient of correlation (*R*^2^) indicates a great similarity between the actual experimental data and the predicted value. The values of *R*^2^, *R*^2^_AdJ._ and *R*^2^_Pred._ have been calculated for the predicted particle size model as 0.9574, 0.9319 and 0.8488, respectively. The proposed model revealed that the smallest particle size of the liposomes (80.6 nm) was achieved when the formulation parameters were as follows: LMC 40 mmole, CH% 20% and DL 3.25 mg/mL. 

### 3.4. Optimized Formulation Parameters for the Lowest PDI

The homogeneity of the liposomal population is important for the biological behavior of liposomes, as large or aggregated liposomes are rapidly cleared from the bloodstream, unlike small uniform ones [39]. The effect of the three selected dependent factors on reducing PDI was studied. The model equation derived for PDI was

PDI = +0.3806 + 0.2445 A + 0.2178 B + 0.0015 C + 0.0698 A^2^ + 0.1298 B^2^ + 0.0288 C^2^

The variance analysis of the regression model for the PDI of the prepared PP-Lip was also checked by the *p*-value and *F*-test (Table 4). The *p*-value of the regression equation was highly significant (˂0.0001) and the lack-of-fit *p*-value was 0.1108 (>0.05). Thus, this fitted regression equation was valid and capable of explaining and predicting the results. Table 4 showed that the individual variables of LMC (A), CH% (B) and the quadratic variable of excess CH% (B^2^) are significant model terms. The values of *R*^2^, *R*^2^_AdJ._ and *R*^2^_Pred._ have been calculated for the PDI regression model as 0.9218, 0.8748 and 0.7158, respectively. The lowest PDI (0.175) was achieved when the formulation parameters were LMC 60 mmole, CH% 20% and drug loading 1.5 mg/mL.

### 3.5. Effect of Individual Variables on the Responses

**Figure 3:**
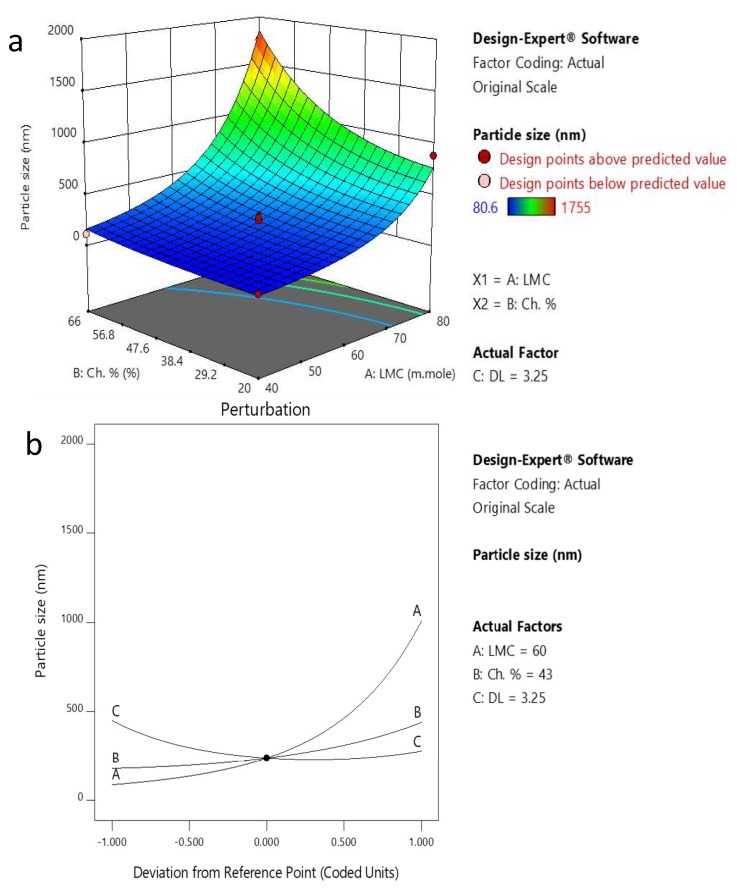
(**a**) 3D plot showing the effect of CH% and LMC on particle size. (**b**) Perturbation blot of factors affecting particle size.

**Figure 4:**
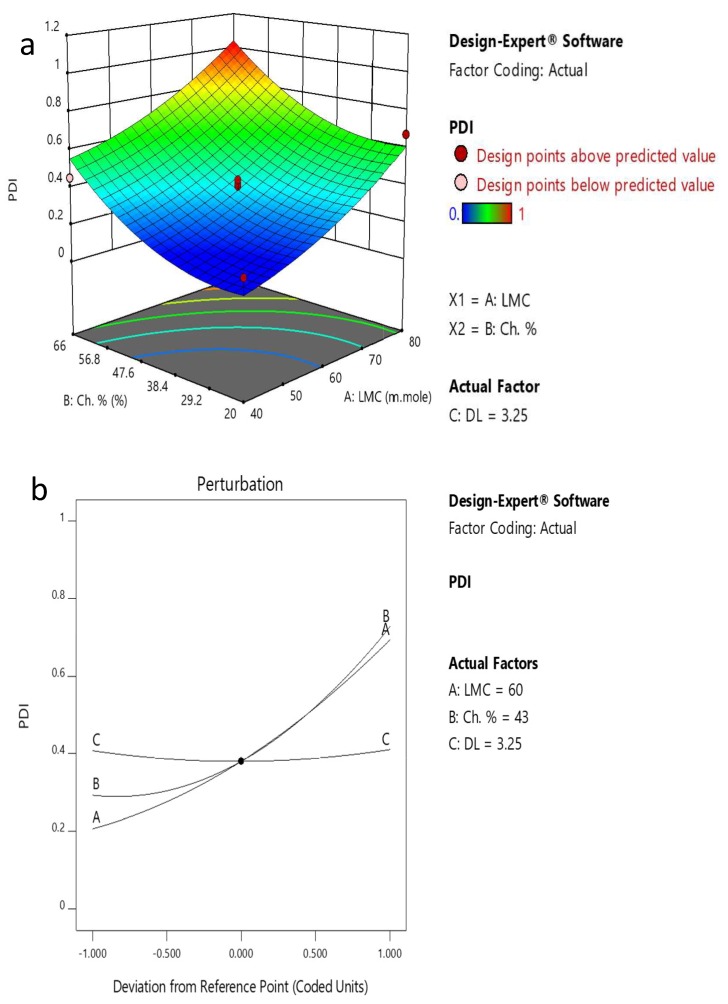
(**a**) 3D plot showing the effect of CH% and LMC on PDI. (**b**) Perturbation plot of factors affecting PDI.

A perturbation plot is used to study the reaction variables’ influence at a particular point in space. The center point of the variables was selected as a constant comparison point between variables. Figure 3a,b) and Figure 4a,b) present these effects on the particle size and PDI, respectively.

LMC (A) has a positive effect on particle size, where increasing the LMC increases the particle size of the formulated liposomes. In addition, increasing CH% (B) slightly increases the particle size while DL (C) negatively affects the particle size.

The 3D plot for factors affecting particle size (Figure 4b) confirms the positive effect of LMC and CH% on particle size, where the particle size is increased approximately 10 times from 80.6 nm to 883.4 nm upon increasing LMC from 40 to 80 mmole at a CH% of 20%. The particle size also increased approximately 17 times from 110 nm to 1755 nm upon increasing CH% from 20% to 66%.

The perturbation and 3D plots of PDI in Figure 3a,b) show that LMC and CH% have strong positive effects on PDI. The PDI increases and the sample becomes more heterogeneous as LMC and CH% increase. On the other hand, the ANOVA test for PDI data supports the suggestion that DL has an insignificant effect on the PDI (Table 4).

### 3.6. Confirming the Optimized Formula with the Optimized Particle Size and PDI 

According to the Box–Behnken design, to obtain the optimized PP-Lip with the smallest particle size and a small PDI value, the formulation parameters of PP-Lip should be set at an LMC of 40 mmole, CH% of 43% and DL of 3.25 mg/mL. At these values, the particle size and PDI presumed by the regression equations were 86.141 nm and 0.216, respectively. At these optimum conditions, five verification experiments were carried out using the spraying technique, and the average particle size and PDI were 90 ± 6.2 nm and 0.23 ± 0.019, respectively. This result confirms that the optimization process was reasonable and feasible. The entrapment efficiency of the optimized formula was measured as a total flavonoidal content and was found to be 57.43 ± 0.09%.

### 3.7. Preparation of the Optimized Formula with TEIM

**Figure 5:**
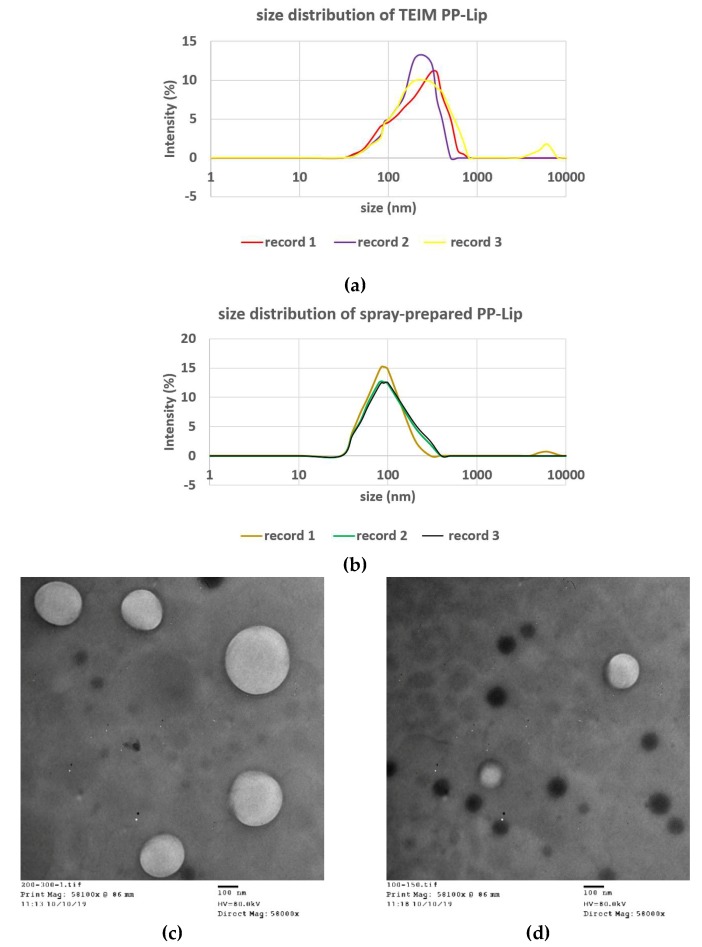
Top: particle size distribution of liposomes prepared using (**a**) TEIM and (**b**) spray method. Transmission electron microscope (EM) image of the (**c**) TEIM-prepared and (**d**) spray-prepared PP-Lip.

The optimum formula was prepared using the typical injection method to set a comparison between the new technique and the typical method of injection in terms of entrapment efficiency (%EE) (Table 5), particle size and PDI. The particle size and PDI were found to be 170 ± 14.7 nm and 0.286 ± 0.017, respectively, when prepared using the TEIM compared with 90 ± 6.2 nm and 0.23 ± 0.019, respectively, with the spraying technique. Figure 5 shows the particle size distribution of PP-Lip prepared using the spray method and TEIM.

### 3.8. In Vitro Cytotoxicity Assay 

**Figure 6:**
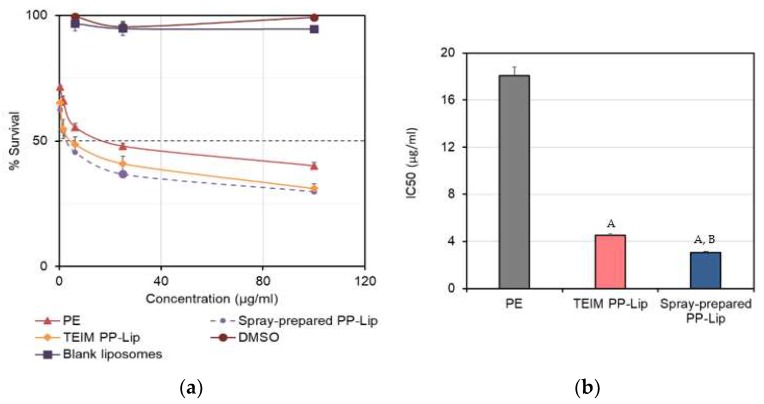
(**a**) Dose–response curves representing the cytotoxic activity of propolis extract (PE), TEIM-prepared PP-Lip and spray-prepared PP-Lip against A375 human melanoma cells, and (**b**) IC50 values (μg/mL) of different formulations. Values are average and *n* = 3. In the bottom figure, A represents a significant difference from PE (*p* < 0.01), and B means significantly different from TEIM PP-Lip (*p* < 0.05).

The anticancer activity of bee propolis has been widely studied in vitro [17,40,41] and in vivo [41,42,43]. A375 cells were treated with spray-prepared PP-Lip, TEIM-prepared PP-Lip and PE. Five concentrations of each sample (100, 25, 6.25, 1.56 and 0.39 μg propolis/mL) were tested to calculate the IC50 and determine cell viability. As shown in Figure 6a, the concentration-dependent cytotoxicity observed with PE was augmented when liposomal formulations were used, as the spray prepared PP-Lip seems to be more cytotoxic than TEIM-prepared PP-Lip. It can be clearly seen that neither the solvent control nor empty liposomes were cytotoxic at the three highest concentrations used. Figure 6b shows that the IC50 values of PE, TEIM-prepared PP-Lip and spray-prepared PP-Lip were 18.06 ± 0.75, 4.51 ± 0.09 and 3.06 ± 0.14 μg/mL, respectively. The one-way analysis of variance shows that all the three groups are significantly different from each other, and that spray-prepared PP-Lip is clearly the most cytotoxic.

## 4. Discussion 

In this research, we hypothesize that formulating propolis into nanosized liposomes will improve its cellular uptake and thus its cytotoxic activity. This can be explained by the fact that liposomes have a better dispersibility and higher surface area for endocytosis. They are able to introduce both hydrophilic and lipophilic components of propolis into the cells inside one carrier. Unencapsulated bee propolis may suffer from precipitation or aggregation in the cellular membrane vicinity due to its lipophilic nature, which may impart poor cellular uptake despite its ability to directly diffuse through the cell membrane. In addition, the water-soluble components may suffer poor cellular uptake because of the lipophilic nature of the cell membrane. On a clinical level, formulating the anti-cancer payload into nano-sized carriers may enable long circulation, which takes advantage of the enhance permeation and retention (EPR) phenomenon that characterizes tumors, especially when these nano-carriers are PEGylated.

The development of proper delivery systems of natural products is highly challenging due to their multi-component nature [44]. Many reports have revealed the cytotoxic action of propolis and its isolated components on various tumor cells [45]. However, the complicated heterogeneous nature of propolis represents a serious obstacle that has impeded the development of a nano formulation with desirable characteristics [11,46]. Liposomes offer the best carrier for such natural products due to their ability to entrap both lipophilic and hydrophilic compounds [44]. Although TEIM has been attempted as a suitable yet economic method for the preparation of most liposomal preparations [38], it has not been shown to be good enough in terms of particle size or size uniformity. The use of TEIM has produced liposomes with an average particle size of about 170 nm; nevertheless, as Figure 4 shows, about half of the liposomes were above 200 nm, and about 25–30% of them were even above 300 nm. This may be attributed to the possible erratic precipitation of various propolis constituents, which leads to an irreproducible distribution within the bilayer membrane. 

We here report a modification of the preparation method that involves a shorter contact time between the ethanolic solution and the aqueous phase, which seems to provide a solution to the above-mentioned problem. This modified method depends on the atomization of an ethanolic solution, containing PE, phospholipids and cholesterol at the surface of the stirred aqueous medium. The spraying technique is an engineered size control tool that provides a homogenous droplet size with a high surface area and enhanced wettability and hydration of the sprayed material [47,48]. The temperature was set at 80 °C, at which propolis was found to be in a molten state [44]. This temperature allows the efficient and reproducible distribution of propolis inside the nanostructure. 

In fact, not only was a markedly smaller particle size obtained with the spray-based method, but also a narrower size distribution, as almost 100% of the liposomes were smaller than 200 nm. Larger and/or aggregated particles may have limited access to cells, compared to their smaller and more uniform counterparts [49,50]. Endocytosis is an energy-consuming process, and the uptake of smaller particles requires less energy than that needed for larger particles [49,51]. Since endocytosis happens over the entire cellular surface, it can be expected that smaller particles which occupy a larger surface area may have a higher chance of cellular uptake [52]. On the other hand, in order to provide more efficient tumor penetration following IV injection of the liposomes, it is strongly recommended that liposomes and nanoparticles are smaller than 200 nm, and preferably 100 nm [53,54]. This highlights the importance of this modified spray-based method, which enabled the encapsulation of a complex mixture of constituents into uniform liposomes with a relatively smaller size. Cell viability was used as a means to detect the efficiency of liposomal uptake, since liposomes made using the two different methods were still composed of the same constituents, in nature and quantities. In general, the propolis-free liposomes were found to be non-toxic to cells at the highest concentration used. Bee propolis extract (PE) itself has a marked antiproliferative activity against A375 cells in vitro. Although TEIM-prepared liposomes showed a significantly lower IC50 value compared to free PE (*p* < 0.01), liposomes prepared using the spray method brought a further reduction of the IC50 value compared to TEIM (*p* < 0.05). These liposomes have a greater potential to enhance the tumor accumulation of PE following IV injection. Our current research is directed towards preparing a long-circulating version of this formulation for tumor treatment.

Response surface methodology is a statistical technique used to mimic the functional relationship between factors and responses by applying multivariable quadratic equations. It is convenient, highly reproducible and highly precise [55,56] and is capable of analyzing each level of the experiment sequentially with a consecutive prediction model [57]. Response surface methodology along with the Box–Behnken design have shown a good predictive power for the optimization of the formulation parameters of the PP-Lip in terms of particle size and PDI. The program revealed that increasing LMC and CH% to 80 mmole and 66%, respectively, resulted in the formation of larger vesicles with a more prominent effect of LMC than CH% (Figure 3 and Figure 4). This is in agreement with Ambardekar et al. [44]. This was attributed to the effect of LMC and CH% on increasing the width and rigidity of the phospholipid bilayer [58].

The results show that particle size of formulated liposomes is inversely proportional to DL. The higher the DL, the smaller the particle size gets. This is inconsistent with Padamwar and Pokharkar, and Bradford et al. [59,60] who stated that increasing the hydrophobic Vitamin E loading up to a certain extent decreased the liposomal size. Above that certain limit, the particle size had a tendency to increase in size, similar to that reported by Ambardekar et al. [44]. Actually, the effect of DL on particle size is difficult to explain due to the numerous components of PE with different polarities and physicochemical characteristics. While increasing the loading of lipophilic drugs that are entrapped within the bilayer usually reduces particle size, increasing the loading of hydrophilic drugs within the inner cavity increases particle size [44]. Here, the presence of many compounds which have different physicochemical properties may lead to an overlapping effect of DL on the particle size. The polydispersity index was found to be higher with increasing levels of both LMC and CH% (Figure 4). Similar findings were reported in [61], [62], and [63]. It is believed that with high LMC, liposomes have a higher tendency for aggregation during preparation [63], which leads to a broader size distribution. The 3D surface plots support the data obtained from the perturbation plots concerning both particle size and PDI and their relation to LMC and CH% (Figure 3 and Figure 4). Accordingly, the optimum preparation conditions based on the Box–Behnken design are LMC 40 mmole, CH% 43% and DL 3.25 mg/mL.

The main advantage of this new method is that it produces smaller and more uniform liposomes using a simple, inexpensive and non-tedious technique. With regard to cancer therapy, a smaller particle size may benefit in increasing cellular uptake and thus anti-proliferative activity and tumor accumulation in vivo (for reasons related to the longer circulation time and taking advantage of the EPR effect). 

For the preliminary in vitro evaluation, we found strong evidence that our approach successfully improved the antiproliferative activity of propolis, either unencapsulated or encapsulated in liposomes, prepared using the more common TEIM method. We believe that this finding accurately met the first part of our hypothesis (with regard to the anti-proliferative activity). We are currently evaluating the second part of our hypothesis, related to tumor accumulation, by taking better advantage of the EPR effect by using the smaller liposomes proposed here for intravenous injection. 

In vivo results will be published as soon as they are finished in a separate publication.

## 5. Conclusions

The spraying technique is more advantageous than the TEIM in the preparation of nanosized homogenous Egyptian PP-Lip with an enhanced cytotoxic effect against melanoma A375 cell line. The new method has the advantages of being simple and saving time and costs with no need for tedious size-controlling methods. 

## Figures and Tables

**Table 1:** Selected factors for the optimization of propolis liposomes (PP-Lip) formulations.

Factors	Codes	Ranges and Levels		
		−1	0	1
Lipid molar concentration (LMC)	A	40	60	80
Cholesterol percentage (CH%)	B	20	43	66
Drug loading (DL)	C	1.5	3.25	5

**Table 2:** Factors and response values for the response surface methodology (RSM) test in preparation of PP-Lip.

Test Number	A (LMC)	B (CH%)	C (DL)	Particle Size (nm)	PDI
1	40	66	3.25	110 ± 19.46	0.45 ± 0.01
2	60	43	3.25	276.6 ± 18.14	0.433 ± 0.038
3	80	20	3.25	883.4 ± 90.06	0.67 ± 0.02
4	60	20	5	189.7 ± 0.49	0.322 ± 0.005
5	40	43	5	85.8 ± 0.58	0.187 ± 0.007
6	80	43	5	1070 ± 111.29	0.749 ± 0.01
7	40	20	3.25	80.6 ± 1.03	0.201 ± 0.009
8	60	20	1.5	241.5 ± 2.39	0.175 ± 0.008
9	60	66	5	665.2 ± 49.12	0.785 ± 0.027
10	80	43	1.5	1678 ± 129.79	0.678 ± 0.02
11	80	66	3.25	1755 ± 194.5	1 ± 0.012
12	40	43	1.5	221 ± 11.53	0.303 ± 0.018
13	60	66	1.5	882.7 ± 274.2	0.875 ± 0.78
14	60	43	3.25	180 ± 18.24	0.41 ± 0.008
15	60	43	3.25	221 ± 14.93	0.37 ± 0.02
16	60	43	3.25	246 ± 6.55	0.29 ± 0.05
17	60	43	3.25	260 ± 10.58	0.4 ± 0.01

LMC: lipid molar concentration, CH%: cholesterol percentage, DL: drug loading.

**Table 3:** Analysis of variance for particle size with the developed model.

Source	Sum of Squares	Df	Mean Square	*F*-Value	*p*-Value	Significance
Model	14.90	6	2.48	37.49	<0.0001	HS
A-LMC	11.80	1	11.80	178.06	<0.0001	HS
B-Ch. %	1.57	1	1.57	23.75	0.0006	HS
C-DL	0.4610	1	0.4610	6.96	0.0248	S
A²	0.2124	1	0.2124	3.21	0.1037	NS
B²	0.1156	1	0.1156	1.74	0.2160	NS
C²	0.6484	1	0.6484	9.79	0.0107	S
Residual	0.6625	10	0.0663			
Lack of fit	0.5409	6	0.0901	2.96	0.1562	NS
Pure error	0.1217	4	0.0304			
Cor total	15.57	16				
*R*^2^ = 0.9574		*R*^2^_Adj._= 0.9319			*R*^2^_Pred._ = 0.8488	

HS: highly significant, S: significant and NS: not significant.

**Table 4:** Analysis of variance for the poly dispersity index (PDI) of the developed model.

Source	Sum of Squares	Df	Mean Square	*F*-Value	*p*-Value	Significance
Model	0.9601	6	0.1600	19.63	<0.0001	HS
A-LMC	0.4782	1	0.4782	58.68	<0.0001	HS
B-Ch. %	0.3793	1	0.3793	46.54	<0.0001	HS
C-DL	0.0000	1	0.0000	0.0022	0.9634	NS
A²	0.0205	1	0.0205	2.52	0.1436	NS
B²	0.0710	1	0.0710	8.71	0.0145	S
C²	0.0035	1	0.0035	0.4292	0.5271	NS
Residual	0.0815	10	0.0082			
Lack of fit	0.0692	6	0.0115	3.75	0.1108	NS
Pure error	0.0123	4	0.0031			
Cor total	1.04	16				
*R*^2^ = 0.9218		*R*^2^_Adj._= 0.8748			*R*^2^_Pred._ = 0.7158	

HS: highly significant, S: significant and NS: not significant.

**Table 5:** Entrapment efficiency (%EE) of the prepared liposomes.

**%EE**	**Spraying Prepared PP-Lip**	**TEIM PP-Lip**
	57.43 ± 0.09%	63.08 ± 1.13%
